# Comprehensive Analysis of Animal Models of Cardiovascular Disease using Multiscale X-Ray Phase Contrast Tomography

**DOI:** 10.1038/s41598-019-43407-z

**Published:** 2019-05-06

**Authors:** Hector Dejea, Patricia Garcia-Canadilla, Andrew C. Cook, Eduard Guasch, Monica Zamora, Fatima Crispi, Marco Stampanoni, Bart Bijnens, Anne Bonnin

**Affiliations:** 1 Paul Scherrer Institut, Villigen PSI, Switzerland; 2 Institute for Biomedical Engineering, University and ETH Zurich, Zurich, Switzerland; 3 PhySense, DTIC, Universitat Pompeu Fabra, Barcelona, Spain; 4 Institute of Cardiovascular Science, University College London, London, UK; 5 Arrhythmia Unit, Department of Cardiology, Hospital Clínic de Barcelona, Barcelona, Spain; 6 BCNatal, Hospital Clínic and Hospital Sant Joan de Déu, Barcelona, Spain; 7 Centre for Biomedical Research on Rare Diseases (CIBER-ER), Hospital Clínic, Barcelona, Spain; 8 ICREA, Barcelona, Spain; 9 Institut d’Investigacions Biomèdiques August Pi i Sunyer (IDIBAPS), Barcelona, Spain; 10 Centro de Investigación Biomédica en Red - Cardiovascular (CIBER-CV), Madrid, Spain

**Keywords:** Image processing, Biomedical engineering, Biological physics, Cardiovascular diseases

## Abstract

Cardiovascular diseases (CVDs) affect the myocardium and vasculature, inducing remodelling of the heart from cellular to whole organ level. To assess their impact at micro and macroscopic level, multi-resolution imaging techniques that provide high quality images without sample alteration and in 3D are necessary: requirements not fulfilled by most of current methods. In this paper, we take advantage of the non-destructive time-efficient 3D multiscale capabilities of synchrotron Propagation-based X-Ray Phase Contrast Imaging (PB-X-PCI) to study a wide range of cardiac tissue characteristics in one healthy and three different diseased rat models. With a dedicated image processing pipeline, PB-X-PCI images are analysed in order to show its capability to assess different cardiac tissue components at both macroscopic and microscopic levels. The presented technique evaluates in detail the overall cardiac morphology, myocyte aggregate orientation, vasculature changes, fibrosis formation and nearly single cell arrangement. Our results agree with conventional histology and literature. This study demonstrates that synchrotron PB-X-PCI, combined with image processing tools, is a powerful technique for multi-resolution structural investigation of the heart *ex-vivo*. Therefore, the proposed approach can improve the understanding of the multiscale remodelling processes occurring in CVDs, and the comprehensive and fast assessment of future interventional approaches.

## Introduction

Most cardiovascular diseases (CVDs) involve cardiac remodelling which is a complex process in which pathological conditions cause a series of genomic expression changes leading to cardiomyocyte death and remodelling, vascular stiffening and dysfunction and fibrosis, among others^1,2^. Without dedicated treatment, the natural evolution of the process leads the heart towards heart failure, with a 50% mortality within 5 years^3^.

In order to better understand cardiac disorders and design appropriate treatments in humans, rodents models of CVDs are often used in preclinical research^4^. In most cases, the goal of such studies is to investigate parameters of cardiac function under differing physiological conditions, ignoring that the key for these changes often lays in the macro- and micro-structural modifications of the tissue. For the purpose of comprehensive structural assessment of cardiac tissue at different length scales, our methodology has been illustrated on several well-known rat models to study remodelling aspects of the myocardium under different CVDs.

For myocardial infarction, isoproterenol treated rats (ISO) are a commonly used model^5,6^. Isoproterenol administration causes an imbalance in the supply-demand of oxygen in cardiac tissue, which leads to an infarction phenotype very similar to that seen in humans, with a marked increase in myocardial fibrosis and extensive scarring^7^.

A second widespread myocardial infarction model is the left anterior descending artery (LAD) ligation model, to simulate myocardial infarction as well as ventricular remodelling and heart failure. The LAD supplies the superior part of the interventricular septum and the superior, lateral and apical wall of the left ventricle. Therefore, after its ligation, these regions are deprived of perfusion, thus leading to (transmural) scar formation^8^.

Another well-known rat model is the spontaneously hypertensive rat (SHR), which is a genetic variation in which continuous hypertension induces the development of heart failure with aging similarly to humans^9,10^.

Up to now, different aspects of these rat models of CVDs (metabolism, function, haemodynamics, structural changes) have been studied by a wide variety of imaging techniques, such as positron emission tomography with X-ray computed tomography (PET/CT)^11^, echocardiography^12^ and magnetic resonance imaging (MRI, including diffusion tensor imaging - DTI)^13–15^. Nevertheless, due to the poor resolution in these techniques, histopathological procedures are commonly required to evaluate the structural tissue characteristics^7–9^.

These studies have brought a specific description of cardiac function and structure at the micro- and macroscale level. However, the complex 3D arrangement of cardiomyocytes, extracellular matrix and microvasculature, their integration into the macroscopic structure as well as its relation with cardiac function are not fully understood. Even less is known about the microstructural remodelling occurring in several CVDs mainly because of lack of non-destructive, 3-dimensional, high-resolution imaging. The use of advanced imaging techniques could provide a deeper understanding of cardiac micro-structure and remodelling, necessary to better assess the influence of CVDs treatments as well as to develop realistic biophysical computational models for future in-silico clinical studies.

Synchrotron Radiation-based X-Ray Phase Contrast Imaging (X-PCI) is a promising imaging technique that can provide the resolution required to accurately visualize overall 3D cardiac morphology and micro-structure at multiscale resolutions. X-PCI takes advantage of the synchrotron X-ray beams’ coherence and the variation in refractive index between different materials in order to improve the contrast while using similar dose deposition compared to synchrotron absorption imaging. Particularly in free-space propagation mode, dose deposition could be greatly reduced thanks to the boost in signal to noise ratio with reduced loss in resolution provided by phase retrieval algorithms^16^, such as the one developed by Paganin *et al*.^17^. Propagation-based X-PCI (PB-X-PCI) has been previously proposed for the assessment of detailed cardiac structure both in excised non-beating rodent^18–20^ and post-mortem human hearts^21–23^. In addition, fetal, neonatal and infant hearts have been assessed using synchrotron grating interferometry (GI)^24,25^, an alternative quantitative phase contrast imaging method. However, this technique would result in more challenging multiscale acquisitions with higher instrumentation requirements. For this proof of concept study, in which high resolution and time-efficiency with a highly coherent source were required, PB-X-PCI was the most suitable technique.

The multiscale cardiac imaging studies present in the literature mainly use optical imaging or MRI. Compared to PB-X-PCI, both techniques are more time consuming and require the use of fluorescent dyes or contrast agents. MRI allows *in-vivo* molecular imaging and fibre tracking by means of diffusion tensor imaging (DTI) but with a lower resolution^26^, while optical methods, such as light sheet fluorescence or episcopic microscopy, are limited by sample size, anisotropic resolution or tissue alterations due to sample processing (slicing, dehydration and use of chemicals among others)^27,28^.

In this study, PB-X-PCI is presented as a 3D quantitative multiscale imaging technique for detailed cardiac tissue analysis. As a proof of concept, four different rat heart specimens, including one healthy and 3 pathological cases (an isoproterenol treated, a LAD ligation and a SHR) were used to evaluate the potential of our proposed methodology to assess cardiac remodelling at both macro and microscopic scales. The proposed multiscale protocol is a methodology where the excised hearts were first imaged entirely at a lower resolution (LR) with 5.8 µm pixel size and then without further specimen manipulation, several small regions of interest (ROI) with an expected remodelling response were selected to be imaged at a higher resolution (HR) with 0.65 µm pixel size. Therefore, PB-X-PCI allows the comprehensive evaluation of cardiac architecture through the assessment of detailed geometrical information of the tissue, based on which microstructural detail can be seen and identified, thus providing similar information as many histological approaches.

This methodology allows the assessment of the overall cardiac geometry, macro-structure and remodelling, with the possibility to also perform a detailed morphological evaluation of the main micro-structures in smaller areas. Different quantitative features can be extracted from the multiscale 3D datasets including: the amount and shape of macro- and micro-vessels, the orientation of bundles of myocytes as well as the amount of extracellular collagen matrix. Moreover, the potential to perform single cardiomyocyte analysis is also shown. The results obtained for the different rat models were compared in order to analyse the differences in cardiac structure present in each of the CVDs models. Finally, the presented methodology is validated by comparison to histological images.

## Results

**Figure 1:**
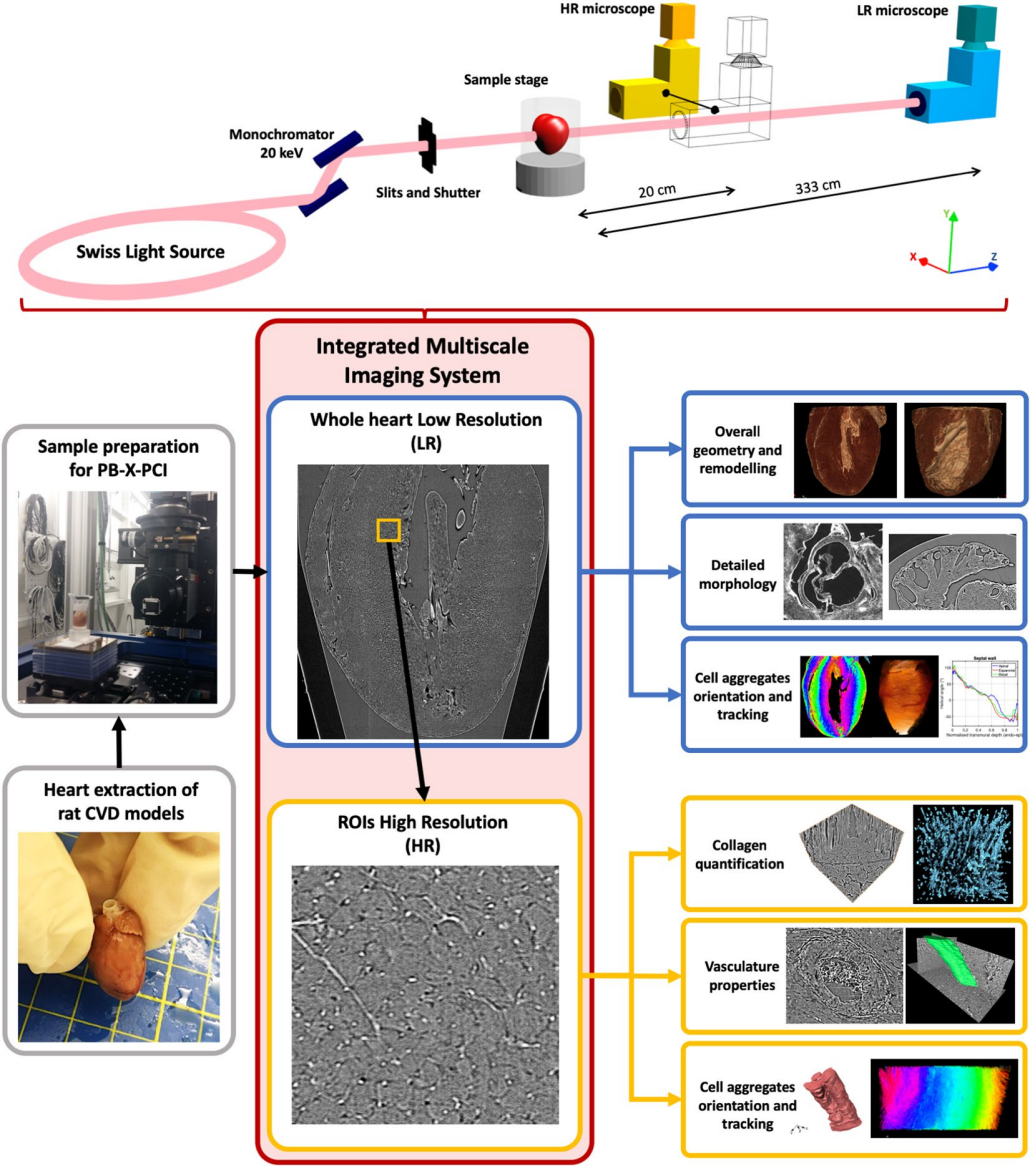
Multiscale imaging and analysis of cardiac tissue. Diagram describing the processing pipeline and imaging setup of the methodology used for comprehensive cardiac tissue analysis.

Figure 1 illustrates our proposed pipeline for the multiscale analysis of rat hearts. LR scans of the whole heart allow the evaluation of the overall cardiac geometry and morphology in 3D, including the estimation of myocyte aggregates orientation. Furthermore, selected ROIs imaged with HR scans can be used to obtain insight into microstructural components, such as collagen, cells and microsvasculature.

To illustrate the different cardiac tissue characteristics that we can quantify, several relevant CVDs models were selected as an example: Wistar Kyoto (WKY) and LAD for the macrostructural analysis and WKY, SHR and ISO for the microstructural assessment.

### Whole heart geometry and detailed morphology

**Figure 2:**
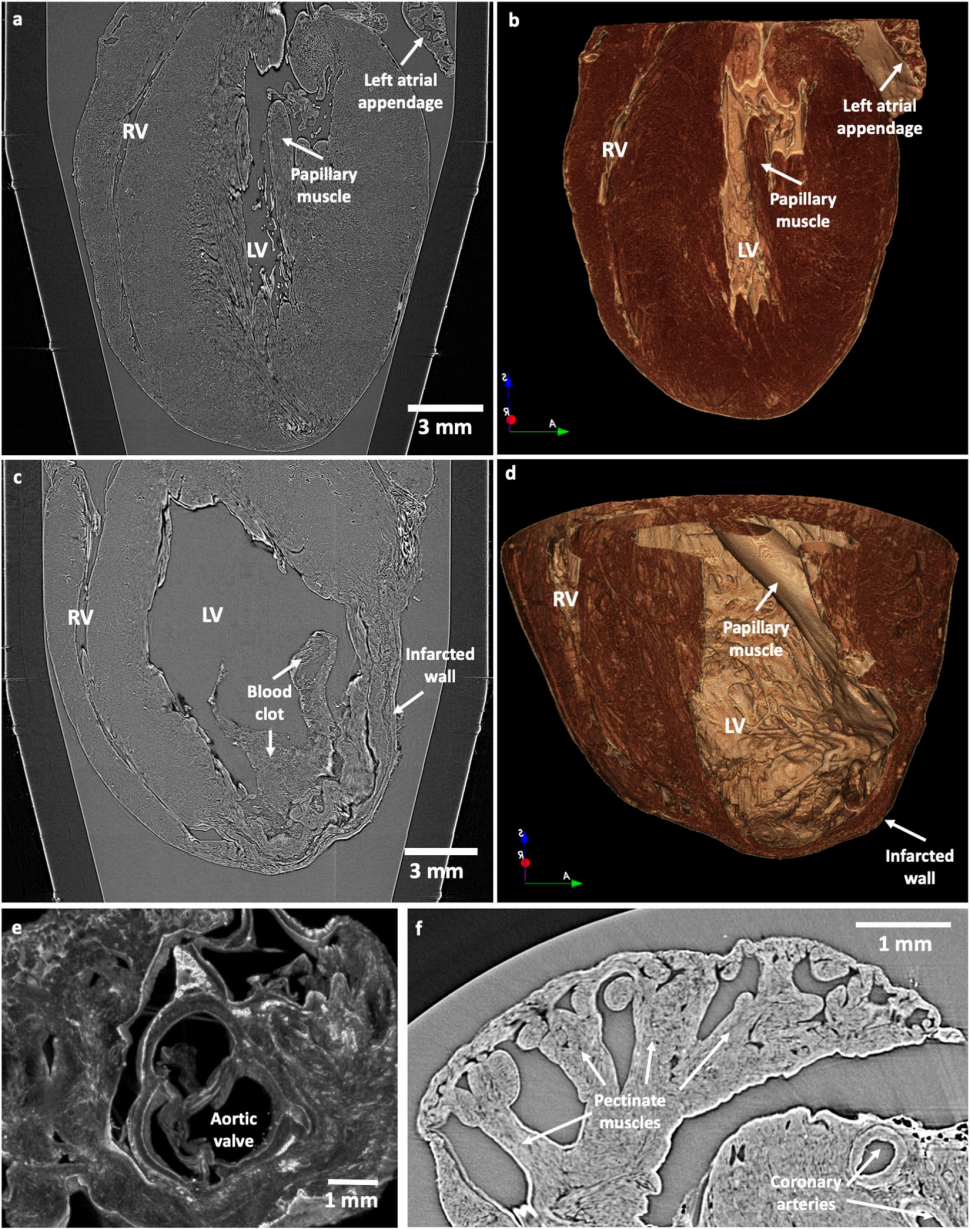
Whole heart geometry and detailed morphology assessment by PB-X-PCI. (**a**) Illustrative longitudinal PB-X-PCI virtual cut of the WKY heart at 5.8 μm pixel size (LR). (**b**) 3D volume rendered image showing the detailed internal structure of both ventricles extracted from the PB-X-PCI dataset in (**a**). (**c**) Illustrative longitudinal PB-X-PCI virtual cut of the LAD heart at 5.8 μm pixel size (LR). (**d**) 3D volume rendered image of the detailed internal structure of both ventricles and the infarcted region, extracted from the PB-X-PCI dataset in (**c**). (**e**) 3D detailed visualization of the aortic valve and surrounding structures in the SHR heart. (**f**) PB-X-PCI image (reconstructed with Paganin’s method) of the pectinate muscles in the left atrial appendage, as well as large coronary arteries in the myocardial wall of the SHR heart.

An illustrative LR PB-X-PCI image of a virtual cut along the longitudinal axis of the WKY and LAD hearts (from base to apex) obtained after image reconstruction can be found in Fig. 2a,c, respectively. In addition, Fig. 2b,d show the corresponding longitudinal virtual cut performed on the 3D volume rendering of the same heart. Finally, Fig. 2e shows a detailed representation of the aortic valve in the WKY heart, and Fig. 2f the left atrial appendage of the SHR heart, where structures such as valve leaflets, pectinate muscles and large coronary arteries can be clearly observed. These figures depict how the LR of the multiscale PB-X-PCI is able to retrieve information from the full heart, which allows to assess overall cardiac shape and structure, as well as remodelling processes affecting the heart at its organ level.

### Myocyte aggregates local orientation quantification and tracking

**Figure 3:**
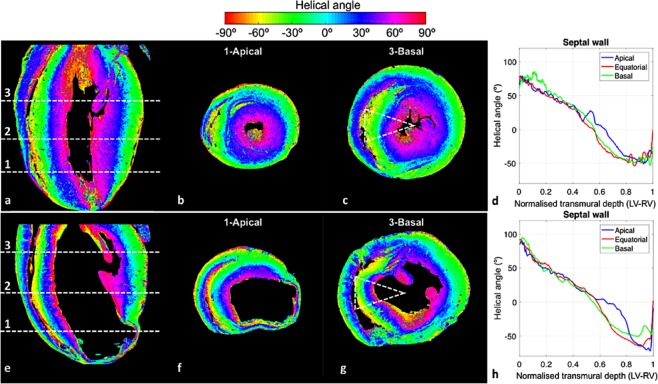
Myocyte aggregates orientation quantification. For WKY (top) and LAD (bottom) hearts, respectively: Ventricular helical angle maps in (**a**,**e**) four chambers view, as well as in (**b**,**f**) apical and (**c**,**g**) basal cross sections slices (marked with dashed white lines in the four chambers views). Colour coded by helical angle values. (**d**,**h**) Transmural profile from left-side endocardium to right-side endocardium of helical angle in the septal wall (dashed triangular area highlighted in **c** and **g**) at three different apico-basal positions (marked with dashed white lines and numbers in the four chambers views: 1-apical, 2-equatorial, 3-basal).

In order to visualise and quantify the orientation of myocyte aggregates (regularly referred to myofibres), ventricular helical angle maps were computed and qualitatively compared, for illustration, in WKY and LAD hearts. Helical angle values were plotted in one longitudinal (four chamber view) and two cross-sectional LR slices at different apical-basal heights as illustrated in Fig. 3(a–c,e,f), where fibre disorganisation can be clearly observed in the infarcted region of the LAD specimen. Images were colour-coded according to helical angle (HA) and circumferential fibres (HA∼0°) are represented in cyan, while longitudinal fibres appear in red. Furthermore, Fig. 3(d,h) shows the transmural profiles from LV endocardium to RV endocardium of HA across the septal wall at three different heights: apical, equatorial and basal slices. The WKY heart shows a normal transmural profile with a gradual change in HA from LV endocardium to RV endocardium as previously described^29^. Nevertheless, in the infarcted heart (Fig. 3h) the slope of the transmural profile of the non-infarcted septum is higher compared to the WKY one (Fig. 3d) and literature describing normal specimens^29^, due to fact that dilatation reorients the LV endocardial myocytes.

### Collagen quantification

**Figure 4:**
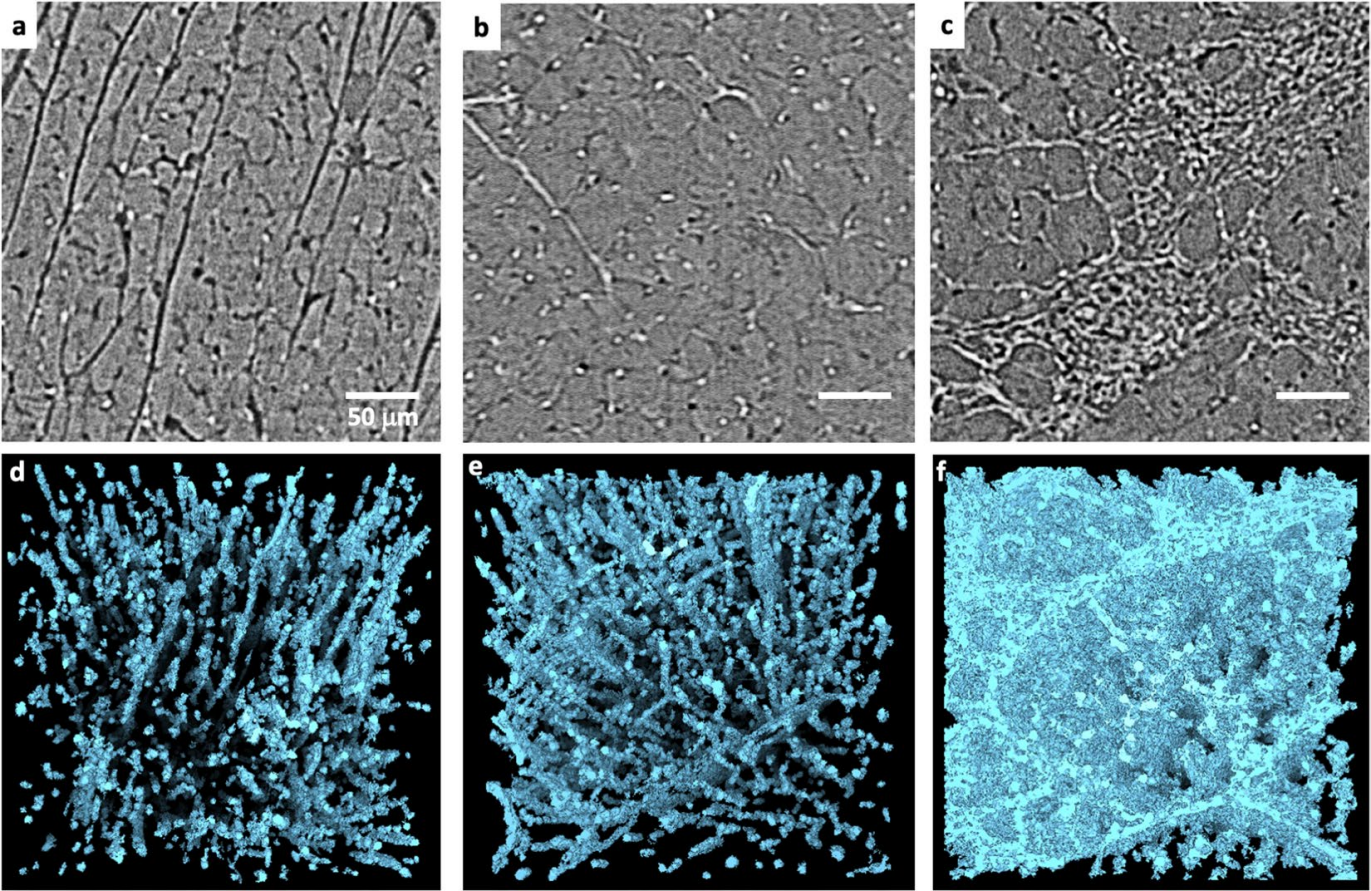
Collagen segmentation in HR images. (**a**–**c**) Representative PB-X-PCI image slices from 300 × 300 × 300 μm subvolumes in the left ventricular septum of the WKY, SHR and ISO hearts respectively. (**d**–**f**) 3D renderings of collagen segmentation in the same subvolumes, visually showing the increase in density and change in shape and distribution around the tissue.

Due to the highest resolution (HR) acquisition and superior contrast, local PB-X-PCI scans allowed us to obtain information on cardiac microstructure. Illustrative slices from different 300 × 300 × 300 μm^3^ subvolumes corresponding to the tomography scans acquired in the LV septum of WKY, SHR and ISO rats are presented in Fig. 4(a–c). Collagen segmentation was performed in the three subvolumes, and 3D volume renderings of the reconstructed collagen matrix are shown in Fig. 4(d–f). It can be clearly observed how its density increases from WKY to SHR, as well as the collagen surrounding the cardiomyocytes leading to the formation of extensive localised areas of scar tissue in the ISO specimen. In the age-matched cases of WKY and SHR, one subvolume of each of the areas scanned at HR was selected to quantify the fraction of collagen with respect to total cellular area. However, since in the case of the ISO and LAD samples the discrimination between collagen among other scar tissue components was challenging, proper quantification could not be done. Furthermore, differences in age would not have allowed for a fair comparison. The computed amount of collagen for the WKY samples was 3.3% (apex), 3.5% (septum RV), 3.5% (septum LV) and 5.4% (lateral wall LV), while for the SHR samples it was 4.4% (apex), 3.4% (septum RV), 4.7% (septum LV) and 9.8% (lateral wall LV). Interestingly, this result shows that collagen percentage increased in LV septum, LV lateral wall and apex, while no changes were observed for the RV septum. In addition, the LV lateral wall presents higher collagen fraction compared to other areas.

### Detailed microstructural components

**Figure 5:**
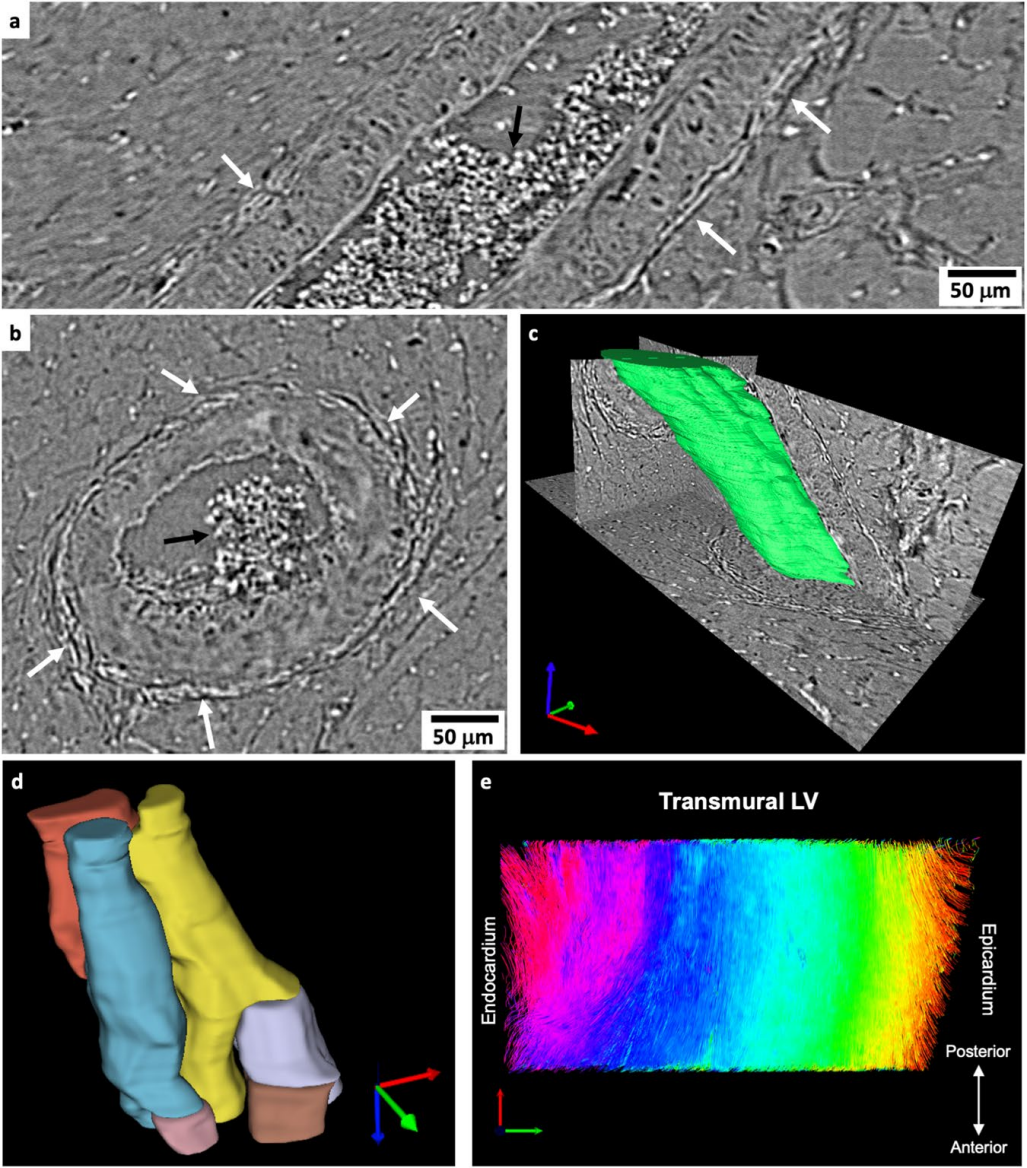
Qualitative analysis of microstructural components of cardiac tissue. (**a**) Longitudinal and (**b**) cross section cuts of a representative coronary artery in the left ventricular (LV) wall of the SHR heart. The white arrows point at the collagen forming the adventitia layer around the vessel wall in the hypertensive rat. Black arrows indicate the presence of blood cells. (**c**) 3D rendering of a section of the visualised artery. (**d**) 3D rendering of a selected bundle of myocytes in the LV of the LAD heart. Colours were changed when branching was observed. (**e**) Fibre tracking in the HR LV transmural volume of the WKY heart. Image was colour coded by helical angle values as in Fig. 3.

Other myocardial tissue structures can be extracted and analysed from the same HR datasets. For illustration purposes, Fig. 5(a–c) depicts an artery from the LV lateral wall of the SHR rat heart, in which the vessel lumen, the arterial wall and the adventitia layer formed mainly by collagen can be distinguished. Moreover, Fig. 5d shows a 3D rendering of a particular bundle of myocytes in the LV wall of the LAD heart. It can be observed with detail how cells branch to connect between each other forming a complex 3D mesh. Colours were changed in every branching point for a better visualization. Finally, local orientation of bundle of myocytes could be assessed also in HR data from a transmural LV volume in the WKY rat as illustrated in Fig. 5e.

### Histological validation

**Figure 6:**
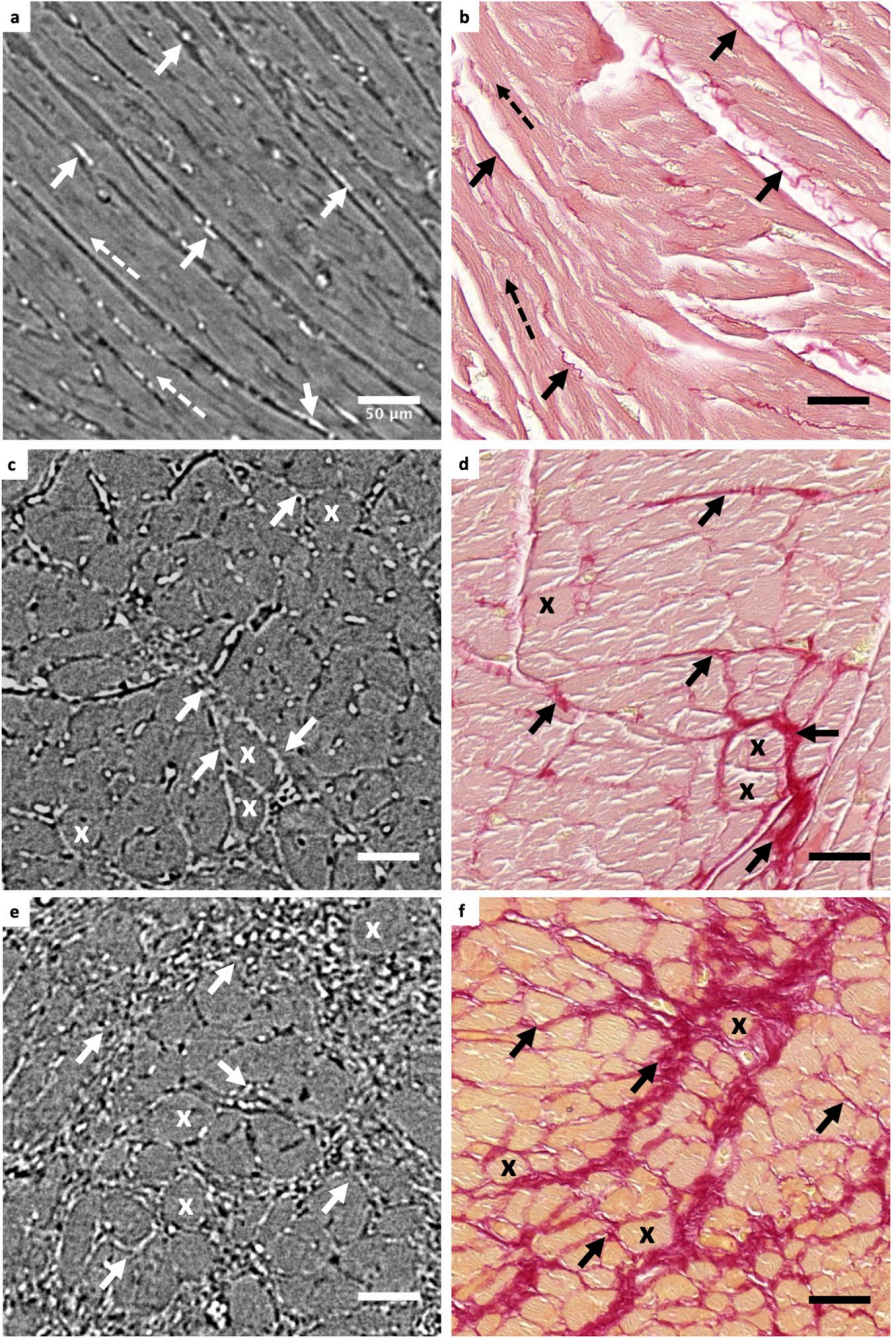
Histological validation of PB-X-PCI. PB-X-PCI images from the apex of (**a**) WKY, (**c**) SHR and (**e**) ISO in comparison with (**b**,**d**,**f**) histological slides stained with Picrosirius red from the approximate same tissue region respectively, with collagen shown in red. Bold arrows indicate the presence of fibrosis, dashed arrows follow longitudinal myocyte direction and crosses mark cross sectional view of the myocytes in both image types.

The structural similarity between the PB-X-PCI images and histology can be observed in Fig. 6. Histological slices from the apex of WKY, SHR and ISO rats were compared to HR images of the same rats and approximately the same region. Similar myocytes oriented longitudinally (dashed arrows) and transversally (X) can be identified in both image types. Furthermore, increased fibrosis was observed in the histological slice of ISO heart and the SHR heart (stained with Picrosirius red), compared to the WKY one, which in PB-X-PCI images is translated to a higher presence of bright matrix-like structures, with similar shape and distribution as observed in the histological images. In WKY, collagen is arranged in thin fibres along the myocytes and in SHR it starts to form sheet-like structures, while in ISO, it forms much larger agglomerations that completely surround the cardiomyocytes, with an appearance of a much more condensed tissue, without intercellular spaces, representing the induced fibrosis. All these features are easily identified both in PB-X-PCI and the histological images.

## Discussion

In this paper, we present a proof of concept of a novel methodology for the comprehensive, fast and quantitative structural evaluation of cardiac tissue, both in healthy and pathological conditions. In order to do so, it is of great importance to study the heart at several scales, allowing assessment of the remodelling in smaller and larger structures, and how all these aspects interrelate. For this purpose, we propose synchrotron PB-X-PCI and a series of image processing tools as a unique technique for the 3D evaluation of cardiac architecture in CVDs. Owing to an integrated multiscale setup with double configuration, whole heart and specific ROIs can be efficiently scanned at 5.8 µm (LR) and 0.65 µm (HR) pixel size respectively, thus allowing the assessment of a wide range of cardiac tissue components at different scales.

First of all, as depicted in Fig. 2, LR acquisitions allow the assessment of overall cardiac geometry due to its larger field of view (FoV), thus being able to look at global/whole organ changes, such as infarct/scar zone, ventricular hypertrophy or dilation. In addition, because of its high resolving power, the morphology of specific internal structures and components, such as valves, false tendons, pectinate muscles, etc. can be visualised with high level of detail (see Fig. 2(e,f)).

In addition, the orientation of the myocyte aggregates was easily computed in LR scans (see Fig. 3). This allows investigation of the organization of myocyte aggregates throughout the heart, and how their predominant orientation is altered under the effects of different CVDs. Particularly, in Fig. 3(e,f) the infarct area located at the LV lateral wall of the LAD heart clearly shows complete fibre disorganisation. In addition, in Fig. 3(d,h) a steeper HA profile of the septal wall can be observed in the LAD case in comparison with the WKY heart.

Moreover, the presented setup can be rapidly switched from one resolution to another. The HR mode with 0.65 µm pixel size, is able to resolve fine components in specific ROIs all across heart. In this context, we demonstrate that a previously developed segmentation pipeline^20^ allows the quantification of the amount of collagen in the tissue and the subsequent comparison between different CVDs models (see Fig. 4), for example, between WKY and SHR. The obtained results showed increased collagen percentage in different LV regions of the SHR compared to WKY, being more significant in the lateral wall, while similar values were observed for RV areas. This is consistent with previous studies performed in the same rat model and ventricular areas^30^. SHR is an inherited hypertensive model that progresses with age towards LV hypertrophy and heart failure^9^ probably caused by renal pro-hypertensive factors^31^, which explains the fact that remodelling and fibrosis development occur only in the LV and not elsewhere.

In addition, other micrometre scale components of cardiac tissue, such as the micro-vasculature and cell aggregates (Fig. 5) can be also assessed in HR datasets. The resolving power of HR acquisitions allows the analysis of single vessels and the detailed visualization of the different vessel wall layers, like the collagenous tunica adventitia, and the possibility to delineate its lumen. Therefore, our proposed methodology could be useful, for example, to study the effect of fibrotic diseases in collagen deposition on the vasculature, or to study other CVDs involving changes in the number of vessels and size of the vascular wall or the lumen itself. The resolution is such that single cell aggregates is made accessible for its assessment.

Furthermore, the helical angle of myocytes aggregates was computed in a transmural LV subvolume from the WKY heart at 0.65 µm pixel size, which, to the best of our knowledge, corresponds to computation of local orientation of myocytes aggregates with highest resolution, thus improving recent efforts with data at 3.5 µm pixel size^23^.

The presented analysis is possible due to the intrinsic nature of PB-X-PCI, which allows multiscale 3-dimensional non-destructive acquisitions of the heart with a superior micrometre level resolution and short acquisition time. In addition, samples did not show any sign of shrinkage, degradation or decolouration after acquisition. Therefore, no observable radiation damage was induced in the tissue.

The combination of our acquisition protocol, together with the post-processing pipelines, have proven useful for the analysis of a wide range of cardiac tissue components at two different resolutions, thus being able to assess microscopic changes and couple them to other macroscopic alterations. As a proof of principle, PB-X-PCI has been used to compare several rat models of CVDs, for which the presented technique has been able to find structural differences compatible with existing reports in the literature.

In this paper, the presented methodology has been shown as a research tool for the study of *ex-vivo* non-beating rat hearts. With the development of new detectors and optics technologies allowing beam enlargement^32^, as well as the increase in computing power, it will open the door to significantly increase the size of the hearts studied.

However, there is limited synchrotron light availability and translation of phase contrast technology to clinics needs to be considered. In this regard, the use of recently developed compact and table top synchrotrons opens the door to the use of propagation imaging^33–35^, since current clinical X-ray tubes do not provide a sufficient photon flux and coherence. Nevertheless, the use of GI would allow the use of these cheaper conventional sources thanks to its lower coherence requirements, but with the aforementioned disadvantage of resolution and flux loss. Promising results have been obtained by X-ray tube-based GI systems in order to translate phase contrast technology to the clinics^36,37^. Both approaches have a lot of potential and the exact application for each of them are being currently investigated.

The cardiac multiscale studies present in the literature focus mainly on MRI and optical imaging. MRI is a complementary technique that potentially presents a better soft tissue contrast with respect to X-ray imaging, as well as spectroscopic and *in-vivo* capabilities. Nevertheless, it is very time consuming for similar resolution requirements and relies in very indirect measurements for tissue structure, while PB-X-PCI directly looks at the physical properties of the tissue (attenuation and refractive index). For instance, MRI often needs the use of contrast agents (usually gadolinium-based) to observe structural changes, such as infarcted zones^26^, and cardiomyocyte aggregates orientation is estimated via DTI, which is an indirect measurement based on the diffusion of water molecules in the tissue. Regarding optical imaging studies, their main disadvantage relies on the sample alteration through tissue processing, the use of fluorescent dyes or staining and tissue slicing. Furthermore, these techniques are time consuming, limited to smaller sample sizes and FoV, as well as anisotropic voxel size^27,28^. Therefore, PB-X-PCI is the only technique able to give insights into both gross anatomy (whole heart morphology and remodelling, cell orientation) and micrometer sized cardiac components (collagen, microvasculature, quasi-individual cardiomyocytes) in 3D, with a faster and easier research pipeline (without tissue processing nor the use of added contrast agents).

Knowing how the microstructural components of the heart arrange, organise and integrate at a macroscopic level is crucial in order to better understand the cardiac (dys)function both in healthy and pathological conditions. Moreover, this information is of prime importance for the development of more realistic and accurate cardiac computational models, for which 3D high-resolution imaging techniques such as PB-X-PCI are needed^38,39^. Therefore, the processing and analysis of images such the ones acquired with the presented protocol, can be used as input for models with the goal of better understanding the effects of anatomical alteration on cardiac function. In addition, PB-X-PCI has the potential to contribute towards the prevention and reversion of cardiac remodelling due to its time-efficient and 3-dimensional characteristics. In this way, the use of PB-X-PCI opens the door for a more comprehensive and complete assessment of dedicated interventional approaches, as well as for the study of the structure-function relationship when used complementarily with *in-vivo* techniques such as MRI or ultrasound imaging.

This study demonstrates that PB-X-PCI is a powerful technique for the *ex-vivo* multiscale analysis of the cardiac structure, allowing the assessment of the different tissue components, from global shape, to myocyte aggregates’ orientation and collagen percentage, thus helping to understand how the microstructural components of the heart integrate into larger structures. In addition, it is presented as a promising protocol for the multiscale study of CVD effects, the differentiation between healthy and abnormal cases, and its use for interventional studies in a faster, 3-dimensional and more comprehensive approach.

## Methods

### Animal models

Four different rat models were selected: an ISO heart of a 2.5 month old male Wistar rat with subcutaneous administration of 2 mg/kg of isoproterenol per day during 4 days, a 1.5 month old male LAD Wistar sacrificed 4 weeks after ligation, a 12 month old male SHR (SHR/KyoRj) and a 12 month old male WKY (WKY/KyoRj) control.

Male wistar rats (250–300 g) used for ISO and LAD were obtained from Charles River Laboratories (Germany and France), while male SHR/KyoRj and WKY/KyoRj were obtained from Janvier Labs (France). The rats were housed individually and maintained at 21 °C with a 12-h day/night cycle. Food and water were administered *ad libitum*.

Animal care and experimentation conformed to the European Union (Directive 2010/63/UE) and Spanish guidelines (RD 53/2013) for the use of experimental animals. Approval was obtained from the local animal research ethics committee “Comité de Ética de Experimentación Animal (CEEA)” (CEEA 68/5435 and CEEA OB533/16).

Details on LAD ligation procedure can be found in the Supplementary Methods.

Following sacrifice of each of the rats, hearts were rapidly excised, rinsed in PBS + 2% heparin solution and immersed in 4% paraformaldehyde. Further ethanol or other chemical treatment was not performed in order to avoid tissue shrinkage and any tissue alteration as much as possible.

For the image acquisition, the hearts were introduced in a cylindrical plastic holder filled with degassed water, specifically designed to hold the heart without compressing it and avoid motion artefacts during tomography acquisition.

### Data acquisition

Synchrotron-based X-ray tomography was carried out at the TOMCAT beamline (X02DA)^40^ of the Swiss Light Source (Paul Scherrer Institute, Switzerland). In order to perform a multiscale study, two different X-ray propagation-based phase contrast imaging microscopes were used (parameters detailed in Table 1).

**Table 1:** Experimental multiscale PB-X-PCI setup specifications.

	LR setup	HR setup
Energy	20 keV	20 keV
Propagation distance	333 cm	20 cm
Effective Pixel size	5.8 µm	0.65 µm
Objective’s magnification	1:1	10x
Field of view	11.83 × 3.29 mm^2^	1.64 × 1.38 mm^2^
Field of view (pixels)	2040 × 578	2560 × 2160
Projections	2501	2501
Darks	20	20
Flats	50	50
Exposure time	20 ms	200 ms
Time per scan	~3 minutes	~11 minutes
Reconstruction time (absorption)	~1 minute	~2 minutes
Reconstruction time (Paganin)	~2.3 minutes	~6.5 minutes
Scintillator	LuAG:Ce 300 µm	LuAG:Ce 20 µm
Camera	PCO.Edge 4.2	PCO.Edge 5.5

First, a “low” resolution (LR) setup was used to acquire a tomographic dataset of the whole heart with 5.8 µm pixel size, FoV of 11.83 × 3.29 mm^2^ and 3330 mm propagation distance. X-rays were converted to visible light through a LuAG:Ce 300 µm scintillator and detected by a sCMOS camera (PCO.Edge 4.2). Even if a detector with 6.5 µm pixel size in chip and a 1:1 microscope was used, pixel size was magnified to 5.8 µm due to beam divergence at 333 cm of propagation distance. Since the hearts were larger than the FoV, scans over 360° were performed to fully cover the heart diameter, and several overlapping scans were acquired vertically to cover the full height. These scans were later stitched in order to obtain full heart datasets. The resulting tomographic dataset was then reconstructed to allow selected regions of interest (ROI) to be imaged with a high resolution (HR) setup with 0.65 µm pixel size, FoV of 1.64 × 1.38 mm^2^ and 200 mm propagation distance. In this case, X-rays were converted to visible light by using a LuAG:Ce 20 µm scintillator, magnified with a 10x objective and detected by a second sCMOS camera (PCO.Edge 5.2). When ROIs larger than a single FoV were selected, overlapping scans were acquired and stitched. An ImageJ script was developed to easily select the HR ROIs from the LR 3D dataset without sample manipulation. In both cases, a 20 keV monochromatic X-ray beam was used. In order to minimise dose deposition^41^, samples were aligned using optical cameras and acquisitions were performed using a 50% power filter (5 mm Glassy Carbon Sigradur), which leads to a flux of 1.3 × 10^11^ photons/mm^2^/s (measured with a PIPS diode^42^). In addition, beam size was adjusted through slits in order to match the FoV and avoid unnecessary tissue exposure.

The acquired projections were reconstructed using the Gridrec algorithm^43^ both in absorption and applying the phase retrieval method by Paganin^17^. The δ/β value used for the Paganin method was finely tuned to 56.9. Illustrative information on scan and reconstruction times can be found in the Supplementary Methods.

### Whole heart rendering and myocyte aggregates orientation quantification

The reconstructed LR absorption datasets were used to segment the whole hearts using *Ilastik*^44^. The resulting segmentations were then loaded in 3DSlicer^45^ in order to render the 3D volume of the heart.

The orientation of the myocyte aggregates was obtained by applying the gradient structure tensor (ST) method^46^ (detailed explanation can be found in the Supplementary Methods) to the same LR absorption datasets. In a last step, the aforementioned segmentations were applied as a mask on the computed HA maps to remove the background and visualize only the results in the cardiac tissue.

### Quantitative collagen segmentation

Following previously described methodology^20^, *Ilastik* was used to label the cardiomyocytes and collagen matrix in representative 300 × 300 × 300 μm^3^ subvolumes, using a combination of the phase retrieved and non-retrieved HR datasets. The procedure starts by iteratively labelling the images for cells, collagen and background in 3D. Since the experimental conditions were the same for all samples, *Ilastik* was able to segment new input datasets with small added supervision. Once the segmentations were complete, they were fused keeping the collagen label from the non-retrieved images and the cell-background label from the retrieved images. This type of fusion was used since, in the case of the non-retrieved data, higher sharpness and collagen contrast is observed while in the phase retrieved case, the contrast cardiomyocyte-background is much higher. With the resulting labelled images, collagen fraction was then computed with respect to cardiomyocyte area.

### Local cell aggregates and microvasculature segmentation

Cell aggregates as well as the lumen of vessel sections were segmented using *3D Slicer*^45^, by manually labelling individual 2D image slices and performing a 3D interpolation. The resulting 3D segmentations were smoothed for better visualization of the volume rendered images.

### Histological validation

Histological validation was performed in the same WKY, SHR and ISO hearts acquired with PB-X-PCI. Following standard histology procedures (Supplementary Table S1), samples were introduced in baths of increasing ethanol concentration (70% to 100%), Xylol and paraffin (Histology Tissue Processor LogosJ PrestoChill, Milestone), embedded in paraffin blocks (Paraffin Embedding Station TES, Medite AG) and sectioned with a microtome (HM 355 S, Microm AG) at 6 μm thickness. Once mounted, the tissue slides were stained using Picrosirius red for collagen identification and imaged using bright field microscopy (Slide Scanner Pannoramic 250, 3D Histech) with 20x magnification (pixel size of 0.38 μm). Histological sections were then matched with PB-X-PCI slices of approximately the same region, since tissue processing and slicing angle during histology hinders a perfect one-to-one matching.

## Supplementary information

Supplementary Material

## Data Availability

Due to the large size of the presented datasets, data will remain stored by the PSI Research Infrastructure following PSI Data Policy (https://www.psi.ch/science/PSIDataPolicyEN/PSI_Data_Policy_201805.pdf). Full accessibility without restrictions will be granted upon request via email to the corresponding Author, including remote access functionality.
