# Author Correction: Comprehensive Analysis of Animal Models of Cardiovascular Disease using Multiscale X-Ray Phase Contrast Tomography

**DOI:** 10.1038/s41598-019-54945-x

**Published:** 2019-11-29

**Authors:** Hector Dejea, Patricia Garcia-Canadilla, Andrew C. Cook, Eduard Guasch, Monica Zamora, Fatima Crispi, Marco Stampanoni, Bart Bijnens, Anne Bonnin

**Affiliations:** 1 Paul Scherrer Institut, Villigen PSI, Switzerland; 2 Institute for Biomedical Engineering, University and ETH Zurich, Zurich, Switzerland; 3 PhySense, DTIC, Universitat Pompeu Fabra, Barcelona, Spain; 4 Institute of Cardiovascular Science, University College London, London, UK; 5 Arrhythmia Unit, Department of Cardiology, Hospital Clínic de Barcelona, Barcelona, Spain; 6 BCNatal, Hospital Clínic and Hospital Sant Joan de Déu, Barcelona, Spain; 7 Centre for Biomedical Research on Rare Diseases (CIBER-ER), Hospital Clínic, Barcelona, Spain; 8 ICREA, Barcelona, Spain; 9 Institut d’Investigacions Biomèdiques August Pi i Sunyer (IDIBAPS), Barcelona, Spain; 10 Centro de Investigación Biomédica en Red - Cardiovascular (CIBER-CV), Madrid, Spain

Correction to: *Scientific Reports* 10.1038/s41598-019-43407-z, published online 06 May 2019

The original version of this Article omitted an affiliation for Monica Zamora and Fatima Crispi.

The correct affiliations for Monica Zamora are listed below:

BCNatal, Hospital Clínic and Hospital Sant Joan de Déu, Barcelona, Spain

Institut d’Investigacions Biomèdiques August Pi i Sunyer (IDIBAPS), Barcelona, Spain

The correct affiliations for Fatima Crispi are listed below:

BCNatal, Hospital Clínic and Hospital Sant Joan de Déu, Barcelona, Spain

Centre for Biomedical Research on Rare Diseases (CIBER-ER), Hospital Clínic, Barcelona, Spain

Institut d’Investigacions Biomèdiques August Pi i Sunyer (IDIBAPS), Barcelona, Spain

Additionally, in the Supplementary information file an affiliation for Eduard Guasch was omitted.

The correct affiliations for Eduard Guasch are listed below:

Arrhythmia Unit, Department of Cardiology, Hospital Clínic de Barcelona, Barcelona, Spain

Institut d’Investigacions Biomèdiques August Pi i Sunyer (IDIBAPS), Barcelona, Spain

Centro de Investigación Biomédica en Red - Cardiovascular (CIBER-CV), Madrid, Spain

This has now been corrected in the HTML and PDF versions of this Article and in the Supplementary Information that accompanies this Article.

