# Supplementary material for: Comprehensive Analysis of Animal Models of Cardiovascular Disease using Multiscale X-Ray Phase Contrast Tomography
Source: Sci Rep. 2019 May 6;9:6996. doi: 10.1038/s41598-019-43407-z (PMC6502928; doi:10.1038/s41598-019-43407-z)
Supplement: Supplementary file 1 — Supplementary Material [file 41598_2019_43407_MOESM1_ESM.docx]

**Comprehensive Analysis of Animal Models of Cardiovascular Disease using Multiscale X-Ray Phase Contrast Tomography**

**Hector Dejea^1,2^*^§^, Patricia Garcia-Canadilla^3,4§^, Andrew C. Cook^4^, Eduard Guasch^5,9,10^, Monica Zamora^6,9^, Fatima Crispi^6,7,9^, Marco Stampanoni^1,2^, Bart Bijnens^3,8#^ and Anne Bonnin^1#^**

**SUPPLEMENTARY METHODS**

**Animal models**

***LAD ligation procedure***

By the LAD ligation, a myocardial infarction was induced in a male Wistar rat as previously described, with minor changes^1^. Briefly, ECG-monitored rats were intubated and ventilated (SAR-1000, CWE Inc) under inhaled anesthesia (isoflurane 2%) and subcutaneous analgesia (buprenorphine 0.05 mg/body kg). Ventilation parameters were set according to the manufacturer instructions, including a 2 cmH_2_O external PEEP. Through a left 4^th^ intercostal incision and thoracotomy, tissue was carefully dissected and the LAD coronary artery exposed at its proximal aspect, in the interventricular septum close to the left atrial appendage. The LAD was ligated with a 6-0 silk; anterior wall myocardial paleness and akinesia were observable immediately in all animals, later followed by overt ST-segment elevation in a DI-like lead. Subsequently, the chest was closed with a 2-0 silk, and the skin with a 3-0 resorbable suture. Recruiting maneuvers were performed before extubating.

***Sample preparation***

Following sacrifice of each of the rats, hearts were rapidly excised, rinsed in PBS+2% heparin solution and immersed in 4% paraformaldehyde. Further ethanol or other chemical treatment was not performed in order to avoid tissue shrinkage and any tissue alteration as much as possible.

For the image acquisitions, the hearts were placed in degassed water and introduced in a cylindrical plastic holder, specifically designed to hold the heart without compressing it and avoid motion artefacts during tomography acquisition.

***Scan times***

As detailed in Supplementary Table S1, LR scans are acquired with 20 ms exposure time per projection, resulting in approximately 3 minutes acquisition time per scan including motor movements and camera read out. Given that several tomograms are needed to cover the full heart, a theoretical rodent heart size of about 18 x 13 x 13 mm^3^ would require a total of 7 scans and approximately 21 minutes acquisition time.

In HR scans, the exposure time was set to 200 ms per projection, resulting in approximately 11 minutes acquisition time, again including the motor movements. For illustration, covering the full left ventricular lateral wall transmurally in a healthy sample (~4 mm) would require 3 volume scans leading to an approximate total acquisition time of 33 minutes.

***Reconstruction procedures and times***

After acquisition, the tomograms were reconstructed using the Gridrec algorithm^2^ in absorption and additionally using the phase retrieval method by Paganin^3^. The Gridrec algorithm is an alternative faster method to conventional filtered back projection based on a regridding procedure used to resample the Fourier space from polar to Cartesian coordinates. Negligible image degradation is observed, mostly in sufficiently sampled scenarios in terms of angular steps, such as here presented.

The single distance phase retrieval method by Paganin is based on the transport-of-intensity equation and consists of a filter applied to the projections in order to retrieve its phase map. The filter response is determined by the δ/β ratio, which corresponds to the ratio between the deviation from 1 of the real part of the sample’s index of refraction, and its imaginary part.

The reconstruction times for single LR volumes were ~1 minute (absorption) and ~2.3 minutes (Paganin), while for the HR scans they are ~2 minutes (absorption) and ~6.5 minutes (Paganin).

**Myocyte Aggregates Orientation Quantification**

A gradient structure tensor (ST) method^4^ was applied to obtain the local orientation of myocytes aggregates. Briefly, the gradient ST analysis consists of the calculation of the oriented intensity gradient in the 3 directions (x, y and z), so that a ST is built for each voxel of the dataset. Then, eigen-decomposition of the ST is performed thus obtaining the three eigenvalues and eigenvectors. The eigenvector with the smallest eigenvalue is selected as the vector pointing in the main direction of the myocyte aggregates, since image intensity is not expected to change along the longitudinal direction of the cardiomyocytes. Finally, the helical angle for the smallest eigenvalue’s vector is calculated as the angle between the transverse plane and the vector projection to the local tangential plane of the cylindrical coordinate system of the heart. Transmural profiles of helical angle within the septal wall were plotted as a function of normalised wall thickness from endo-to epicardium (or right-side endocardium).

Finally, 3D fibre tracking was performed in the whole heart along the eigenvector with smallest eigenvalues using a 2^nd^order Runge-Kutta method in Paraview^5^. Tracks were colour-coded by transmural depth.

**SUPPLEMENTARY TABLES**

**Supplementary Table S1: Experimental multiscale X-PCI setup specifications.**

|  | LR setup | HR setup |
| --- | --- | --- |
| Energy | 20 keV | 20 keV |
| Propagation distance | 333 cm | 20 cm |
| Effective Pixel size | 5.8 µm | 0.65 µm |
| Field of view | 11.83 x 3.29 mm^2^ | 1.64 x 1.38 mm^2^ |
| Projections | 2501 | 2501 |
| Darks | 20 | 20 |
| Flats | 50 | 50 |
| Exposure time | 20 ms | 200 ms |
| Time per scan | ~3 minutes | ~11 minutes |
| Reconstruction time (absorption) | ~1 minute | ~2 minutes |
| Reconstruction time (Paganin) | ~2.3 minutes | ~6.5 minutes |
| Scintillator | LuAG:Ce 300 µm | LuAG:Ce 20 µm |
| Camera | PCO.Edge 4.2 | PCO.Edge 5.5 |

**References**

1. Cardin S, Guasch E, Luo X, Naud P, Le Quang K, Shi Y, Tardif J-C, Comtois P, Nattel S. Role for MicroRNA-21 in Atrial Profibrillatory Fibrotic Remodeling Associated With Experimental Postinfarction Heart Failure. *Circ Arrhythm Electrophysiol*. 2012;5:1027–35.

2. Marone F, Stampanoni M. Regridding reconstruction algorithm for real-time tomographic imaging. *J Synchrotron Radiat*. 2012;

3. Paganin D, Mayo SC, Gureyev TE, Miller PR, Wilkins SW. Simultaneous phase and amplitude extraction from a single defocused image of a homogeneous object. *J Microsc*. 2002;

4. Baličević V, Lončarić S, Cárdenes R, Gonzalez-Tendero A, Paun B, Crispi F, Butakoff C, Bijnens B. Assessment of myofiber orientation in high resolution phase-contrast CT images. In: Lecture Notes in Computer Science (including subseries Lecture Notes in Artificial Intelligence and Lecture Notes in Bioinformatics). 2015.

5. Ahrens J, Geveci B, Law C. ParaView: An end-user tool for large-data visualization. In: Visualization Handbook. 2005.
